# Knowledge gaps impeding plastic governance in the European Union

**DOI:** 10.1016/j.isci.2026.117065

**Published:** 2026-08-13

**Authors:** Stine Hach Juul Madsen, Fredric Bauer, Teis Hansen, Lars J. Nilsson, Bethanie Carney Almroth, Jonathan M. Cullen, Elin Dreyer, Leonidas Milios, Lars Fogh Mortensen, Tobias Dan Nielsen, Tara Olsen, Kristian Syberg, Arnold Tukker, Esther van den Beuken, Patricia Villarrubia-Gómez

**Affiliations:** 1 Department of Food and Resource Economics, University of Copenhagen, Rolighedsvej 23, Frederiksberg C 1958, Denmark; 2 Department of Technology and Society, Lund University, PO Box 118, Lund 22100, Sweden; 3 SINTEF, Department of Technology Management, S.P. Andersens Veg 5, Trondheim 7031, Norway; 4 Department of Biological and Environmental Sciences, University of Gothenburg, Gothenburg, Sweden; 5 Department of Engineering, University of Cambridge, Trumpington Street, Cambridge CP2 1PZ, UK; 6 Joint Research Centre of the European Commission, Calle Inca Garcilaso, Seville 41092, Spain; 7 European Environment Agency, Kongens Nytorv 6, Copenhagen K 1050, Denmark; 8 Department of Science and Environment, Roskilde University, Universitetsvej 1, Roskilde 4000, Denmark; 9 Institute of Environmental Sciences (CML), Leiden University, Einsteinweg 2, Leiden 2333 CC, the Netherlands; 10 TNO Vector, Anna van Buerenplein 1, Den Haag 2595 DA, the Netherlands; 11 TNO Unit Energy and Material Transition, Kessler Park 1, Rijswijk 2288 GS, the Netherlands; 12 Stockholm Resilience Centre, Stockholm University, Stockholm 106 91, Sweden

**Keywords:** plastic governance, knowledge gaps, systems approach, strategic action

## Abstract

Plastic pollution poses urgent environmental, societal, and health challenges, yet governance remains fragmented. The 2022 UNEA resolution 5/14 offers an opportunity for a legally binding treaty, but negotiations are stalled by geopolitical tensions, entrenched industrial interests, and divergent views on the plastic problem. Effective governance is further constrained by knowledge gaps spanning the entire plastic life cycle. The European Union, as a leading proponent of life-cycle-oriented plastic governance, provides a relevant context to examine these gaps. This paper offers an analysis of critical knowledge gaps that impede effective plastic governance across plastic life cycles. Through a multi-stage process drawing on structured expert input and supporting review of relevant literature, we identify four strategic action areas essential for progress in the European Union: knowledge generation, methodological development, transparency and disclosure, and tracking and traceability. Advancing these areas is important to addressing the urgent and complex challenges posed by plastic pollution.

## Highlights

• Thirteen knowledge gaps impede effective EU plastic governance
• Four strategic action areas address key governance barriers
• Transparency and traceability are critical for policy effectiveness
• Missing standards and disclosure constrain policy action

## Graphical abstract

**Figure:**
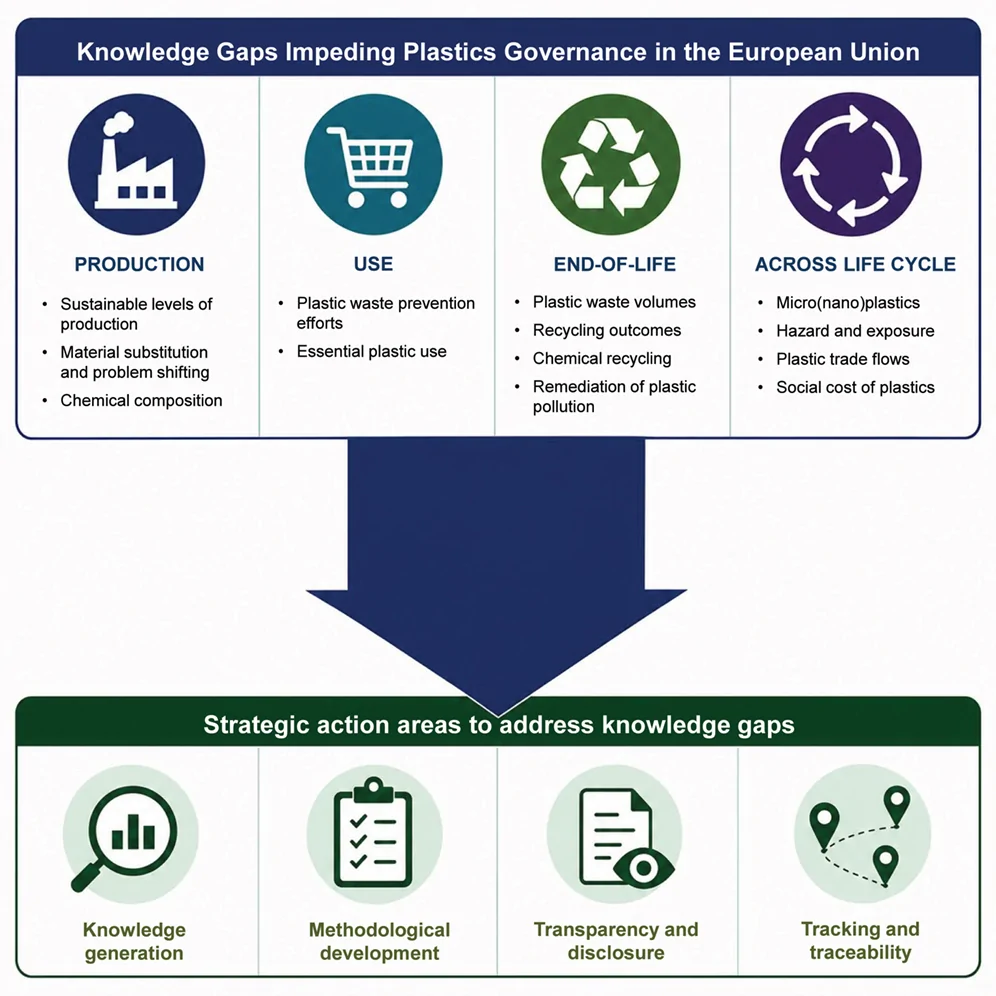

## Teaser

Environmental science; Interdisciplinary application studies

## Introduction

The global challenges posed by plastic pollution have emerged as a critical environmental and societal concern of the 21st century.^1^ Recent research demonstrates that plastic pollution aggravates the impacts on all planetary boundaries,^2^ and substantial evidence documents the adverse impacts of plastic pollution on human health, ecosystems, and biodiversity.^3^^,^^4^ This helps explain the growing attention toward plastic governance across academic, policy, and industry spheres. This growing attention is reflected in a proliferation of governance initiatives at multiple scales.

Perhaps most prominent is the multilateral effort to secure an international legally binding instrument to eliminate plastic pollution. The 2022 United Nations Environment Assembly (UNEA) resolution 5/14, mandating treaty negotiations, represents a critical opportunity to address the issue of plastic pollution systemically, moving beyond the historically fragmented approach of targeting individual environmental domains, product categories, or waste streams.^5^ While earlier interventions primarily focused on specific items such as plastic bags or single-use products,^6^ the UNEA resolution recognized that plastic pollution occurs from production and transportation to waste handling and mismanagement. Consequently, it acknowledges that effective governance requires a systemic approach, addressing the full life cycle of plastic feedstocks, products, and wastes.

Despite the initial recognition of the systemic nature of plastic pollution, the inconclusive treaty negotiations during the past three years reveal deep-rooted tensions between nations that frame the plastic challenge as primarily a waste management issue and nations that continue to insist the challenge must be addressed across the life cycle of plastics.^7^ This highlights the very challenging task of landing an ambitious global treaty on an issue with such strongly vested interests in the current geopolitical landscape.^8^ These interests reflect not only divergent policy preferences but also profound carbon lock-ins in the petrochemical-based plastics industry, which through technological, institutional, and behavioral phenomena shape the political feasibility of far-reaching life cycle interventions.^9^^,^^10^

However, while political will and vested interests are decisive factors shaping the scope of a future international agreement, the ability to govern plastics effectively is also constrained by substantial knowledge gaps (KGs) that persist across the life cycle of plastic.^11^^,^^12^^,^^13^^,^^14^ These gaps span production, use, end-of-life, and cross-cutting issues, and they encompass not only technical and scientific uncertainties but also social, political, and economic dimensions. The complexity of these KGs is further amplified by the rapid evolution of plastic materials, changing consumption patterns, and recycling challenges, all of which create uncertainty and shifting conditions for policy interventions.

Against the backdrop of the current global political deadlock, we present an analysis of key KGs that constitute barriers to effective plastic governance, accompanied by an action agenda for addressing these gaps. The analysis draws on a structured expert workshop and a synthesis of relevant literature focused on the institutional setting and governance legacy of the European Union (EU). The EU constitutes a relevant case given its ambition to advance life-cycle-oriented approaches to plastic governance, most notably articulated in the 2018 European Strategy for Plastics in a Circular Economy.^15^ In the context of the ongoing international treaty negotiations, the EU has further positioned itself as a leading proponent of ambitious global action, including through its participation in the High Ambition Coalition.^16^ As one of the actors likely to champion and implement ambitious provisions under a future global plastics agreement, the EU’s ability to address these KGs will be central to the credibility and effectiveness of such an instrument. This analysis thus proceeds from the assumption that addressing KGs can enable, but not on its own guarantee more effective plastic governance in a context shaped by strong political and economic constraints.^12^

Our analysis highlights that KGs are not the result of missing evidence alone. Rather, they also stem from the absence of standardized methodologies, limited transparency and disclosure, and insufficient systems for monitoring and tracing plastics across their life cycle. In this sense, KGs do not merely coexist with political deadlock; they also shape what can be proposed, implemented and enforced, and as such matter for the scope and legitimacy of plastic governance. This has important implications for how such gaps can be addressed. Our findings indicate that many of the identified gaps cannot be resolved through further research alone, but require interventions in how knowledge is generated, standardized, disclosed, and made accessible within governance systems. By identifying these KGs and linking them to concrete action areas, this study moves beyond mapping missing knowledge toward understanding how constraints can be addressed, providing actionable insights for researchers, policymakers, and practitioners working toward more sustainable plastic systems.

The remainder of the article is structured as follows. Section 2 describes the research process and methodology. Section 3 presents the results, identifying and characterizing key KGs and analyzing how these gaps impede effective governance across the plastics value chain, including production, use, end-of-life management, and cross-cutting life cycle themes. In section 4, we translate these findings into four strategic action areas to support more effective plastic governance, explicitly linking each action area to the KGs it addresses. Section 5 concludes by outlining key takeaways.

## Results

### KGs related to plastic production

#### KG 1.1 Sustainable levels of production

Defining sustainable production levels for plastics remains a difficult task due to the complex environmental impacts associated with the feedstock extraction of raw materials and primary plastic production, including air and water emissions that affect frontline communities and nearby ecosystems, greenhouse gas emissions, and plastic losses during production.^17^^,^^18^^,^^19^ Data about primary plastic production are opaque; environmental impact databases carry significant uncertainties due to variations in feedstock and processes, and there is no transparent reporting of these activities.^20^ While global plastic production continues to increase, we lack robust frameworks for determining production thresholds that remain within the carrying capacity of critical Earth systems, as well as a widely accepted reference baseline of impacts associated with production. While defining a sustainable level of production is not solely a scientific task but also requires societal choices about the impacts of a production restriction on both ecosystems and economies; evidence strongly points to the need for reducing production.^21^^,^^22^ This is also recognized by more than 100 states, including the EU member states, that have supported the inclusion of production restrictions in the plastic treaty negotiations, showcasing the political relevance for this type of knowledge. Expanding the knowledge base and valuation procedures for defining sustainable plastic production is necessary for plastic governance to adequately protect our climate and ecosystems.

#### KG 1.2 Material substitution and problem shifting

As pressure mounts to find alternatives to petrochemical plastics, there is insufficient understanding of when and how substitution leads to unintended consequences or merely shifts environmental burdens, e.g., higher climate impact for specific products and applications, or conflicts over land and water use.^23^^,^^24^^,^^25^ Without standardized assessment methodologies for comparing petrochemical plastics to alternatives (e.g., biobased, biodegradable, whether biobased or petrochemical-based, and other substitutes including non-material-based systems and infrastructure) across diverse applications and contexts throughout their life cycle, decision makers lack the evidence base needed for sound material selection policies. While life cycle assessment (LCA), a method intended to evaluate the environmental impact of a product from production to disposal, is expected to play a central role in this process, current LCA methodologies remain underdeveloped.^26^ Consequently, policymakers risk promoting alternatives that may have worse overall environmental impacts than the plastics they replace.

#### KG 1.3 Chemical composition

There is limited transparency regarding the chemical composition of plastic products. With thousands of additives used in plastic manufacturing and limited disclosure requirements, critical information about the chemical compounds used in plastic products is not disclosed or available to regulators, recyclers, and citizens.^27^^,^^28^ This lack of transparency is compounded by the presence of non-intentionally added substances (NIAS), which are by-products from manufacturing and degradation, or sorbed during use.^29^ Because the identity, concentration and formation pathways of NIAS are often unknown and can vary, depending on, for example, manufacturing conditions or product use, they present an additional layer of uncertainty.^30^^,^^31^

This opacity undermines governance efforts aimed at ensuring product safety and enabling greater plastic circularity (cf. hazard and exposure). Without comprehensive knowledge and transparency about plastic composition, policymakers cannot develop targeted regulations for substances of concern, recyclers cannot establish appropriate sorting and processing techniques, and citizens cannot make informed choices. This KG also complicates efforts to establish circular economy systems, as unknown additives and contaminants can render recycled materials unsuitable for high-value applications.

### KGs related to plastic use

#### KG 2.1 Plastic waste prevention efforts

Methods to measure and monitor plastic waste prevention are underdeveloped,^32^^,^^33^^,^^34^ which means it is unclear how much plastic waste is avoided through efforts to extend product lifespans, that is, the period a product remains in active use.^34^ Such efforts include, for instance, reuse, where products are used again for their original purpose as well as repair, which extends the functionality of the product.^34^ While some EU member states quantify the reuse of selected product groups, there is currently no national monitoring across a broad set of product categories. Moreover, to contribute to waste prevention, reuse should replace the purchase of a new product. However, information on product-specific replacement rates is not available, which means we cannot assume that data on reuse reflect actual waste prevention.^32^

This KG hinders the institutionalization of policy efforts to prevent plastic waste, as the lack of data inhibits both the assessment of progress and the evaluation of the impact of current waste prevention policies.^35^ Furthermore, a better understanding of how actual product lifespans compare to the lifetime a manufacturer intends its product to remain functional, the so-called designed lifetime, would be useful to evaluate and prioritize policy interventions aimed at extending product lifespans.^34^

#### KG 2.2 Essential plastic use

While plastic use has expanded across all sectors and systems of society, with projections indicating continued and substantial growth in production and consumption,^36^ there remains no broadly agreed and operationalized framework for assessing the essentiality of plastic use and polymers across different contexts, including for prioritizing uses^37^ and phasing out of hazardous plastic chemicals.^38^ Although essential-use approaches have begun to emerge in EU chemicals policy,^39^^,^^40^ important conceptual and implementation gaps remain. A key challenge in this regard is that essentiality is inherently context-specific and time-bound, varying across regions depending on socioeconomic conditions, infrastructure, and the availability of technically and economically feasible alternatives.

In the absence of context-sensitive criteria for assessing essentiality, decision makers in the EU as well as in other regional contexts, lack the necessary guidance to eliminate or reduce risks from non-essential plastics and hazardous plastic chemicals. This further constrains the development of policies that ensure feedstock is allocated to essential uses, such as the transition to green energy and applications essential for healthcare. Moreover, determining essentiality must necessarily be linked to an understanding of the plastic production volumes society can sustainably manage (cf. sustainable levels of production).

### KGs related to the end-of-life of plastics

#### KG 3.1 Plastic waste volumes

EU waste statistics do not fully account for the volume of plastic waste. Plastics ending up as litter is not accounted for, as the plastic is not collected. Data on plastic waste in non-packaging waste streams are lacking or are of lower quality and comprehensiveness, although they represent most of the European plastic consumption.^41^ Finally, waste composition analyses of residual waste identify additional plastics that are not included in recorded plastic waste volumes.^42^^,^^43^

This incomplete picture results in an overestimation of plastic recycling rates in the EU,^44^ which hampers assessment of policy progress. Moreover, the limited data on non-packaging plastics hampers investment decisions in future plastic waste management infrastructure by making it difficult to assess what technologies and capacity are needed. For example, without reliable estimates of how much durable plastic from sectors such as electronics or construction enters the waste stream, it is difficult to determine whether investments in specialized sorting or advanced recycling facilities are warranted.^41^

#### KG 3.2 Recycling outcomes

In the EU, little data are available on how, when, and where mechanically recycled plastic materials are used, even for the most strictly controlled fraction of PET bottles. In part this KG results from restricted access to verifiable primary industry data. Consequently, it is hard to track the quality of plastic recycling and degradation over multiple recycling stages, as well as whether recycled plastics substitute primary materials.^45^^,^^46^ These partial understandings hamper the assessment of policy impact and progress and make it particularly difficult to assess the human health, climate, and environmental impacts of recycling.

#### KG 3.3 Chemical recycling

Multiple uncertainties and a lack of research, knowledge, and understanding prevail regarding chemical recycling and the technologies used for it, including their effects on the environment and climate, technical and economic feasibility, and industrial-scale performance and outcomes (proportion of monomers, base chemical products, and fuel).^47^^,^^48^^,^^49^ These uncertainties are, in part, linked to a lack of transparency in the chemical recycling sector, where public access to verifiable evidence supporting industry claims of viability remains limited.^50^^,^^51^^,^^52^

Consequently, there is sparse knowledge to base regulation on, which is particularly concerning given that public funds are already being spent,^53^ and investments in these technologies increased almost 10-fold from 2019 to 2023, reaching more than 600 million EUR in the EU.^54^ In addition, little is known about potential human health implications for workers in chemical recycling facilities, reflecting a broader KG in occupational health in the EU waste sector.^55^

#### KG 3.4 Remediation of plastic pollution

Knowledge is missing on the most suitable technologies and methods for the remediation of plastic pollution across different natural environments. Numerous plastic remediation technologies have been developed, some aimed at preventing more plastics from entering aquatic environments, others targeted at cleaning up existing plastics from the environment.^56^ While a range of plastic remediation technologies exist, their effectiveness, potential unintended consequences, and trade-offs are not well known.^57^^,^^58^^,^^59^ Research also questions the scale of impact of current remediation efforts under the ongoing increases in virgin plastic production^60^ and points to challenges for remediated plastics to re-enter the economy due to low recycling potential and high transportation and energy costs.^58^ These uncertainties complicate decisions on prioritizing support and investment for remediation efforts. Even with an immediate halt to plastic pollution, existing pollution in ecosystems would remain a major challenge, emphasizing the need to better understand the potential of remediation strategies.

### KGs across the plastics life cycle

#### KG 4.1 Micro(nano)plastics

Microplastics, commonly defined as plastic particles between 1 μm and 5 mm, and nanoplastics, typically considered to be in the size range 1–1000 nm,^61^ are an emerging concern as the sources and extent of micro(nano)plastic leakage as well as the prevalence and fate of particles remain a significant KG. We lack data on the release of primary and secondary (formed via fragmentation, mechanical abrasion, heat/UV irradiation, etc.) micro(nano)plastics during all stages of the plastic life cycle. Current research highlights significant contributions from sources, including pellets (early in the life cycle, pre-product manufacturing) or fibers during production, shedding during use (textiles, car tyres, packaging, and paint) and later during recycling or end-of-life, but no consolidated overview exists. Although larger-size microplastics are more extensively documented, understanding across the full-size range is still limited.^62^^,^^63^^,^^64^ In addition to these source-related uncertainties, major gaps remain regarding the prevalence and fate of micro(nano)plastics in the ecosystems they end up in.^65^^,^^66^

Without better knowledge of micro(nano)plastic sources, as well as their prevalence and fate, interventions to minimize leakage and assess exposure to hazardous substances are unlikely to be effective (cf.hazard and exposure). The recently approved EU regulation on preventing plastic pellet losses illustrates how scientific knowledge on microplastic pollution can directly inform targeted policy measures. In this case, the regulation is explicitly motivated by evidence showing that “plastic pellet losses constitute the third largest source of microplastics unintentionally released to the environment in the Union”.^67^ This illustrates how identifying, in this case sources, can support the motivation for targeted policy.

#### KG 4.2 Hazard and exposure

There is a considerable KG related to the hazard of, and exposure to plastic chemicals and particles for humans and the environment, which impedes the ability to assess and manage the risk of plastics. A substantial portion of plastic chemicals lack hazard information,^68^^,^^69^ meaning it is currently unknown whether many chemicals present in plastics have the intrinsic potential to cause harm to human health or the environment.^70^ Furthermore, interactions among chemical additives in plastics are unknown in most cases, so potential cocktail effects are not adequately accounted for in current hazard and exposure assessments.^71^

This KG is linked to methodological challenges. For example, for plastic particles^72^ methodologies for identifying and quantifying nanoplastics remain insufficiently developed,^72^^,^^73^ and existing toxicity tests, developed for soluble chemicals, are not necessarily well-suited to assess particle toxicity.^74^ Similarly, robust data on exposure to plastic chemicals, which take mixture toxicity and NIAS into account, are not available and difficult to collect.^75^^,^^76^

This KG hinders the development of science-based safety thresholds, leaving uncertainty around acceptable levels of exposure to plastic chemicals and particles for both humans and the environment. The lack of knowledge is compounded for recycled plastics because the source material is heterogeneous and not easily determined.^31^^,^^77^^,^^78^ Related uncertainties also remain regarding how repeated use and material degradation in reuse systems may influence chemical exposure over time.^31^^,^^79^ Together, these KGs hamper the ability to make informed decisions about the appropriate use of circular plastic applications, which is particularly alarming considering the strong policy push in the EU to increase plastic circularity, noticeably through achieving higher recycling rates.^15^

#### KG 4.3 Plastic trade flows

A significant KG exists regarding the movement of plastics that remain within the economic system. The main challenge in understanding plastic trade flows is capturing the full spectrum of plastic materials and their life cycle stages in official statistics.^80^^,^^81^ Upstream, global primary plastic trade is estimated at over 1 trillion USD annually.^82^ However, this figure still underestimates the full scale of the global plastic trade due to “hidden” plastics embedded in various products and packaging such as electronics and textiles.

Downstream, multiple challenges co-exist that prevent reliable data on plastic waste flows. First, while trade statistics like UN COMTRADE offer insight into the legal trade of waste plastics,^83^ underreporting again follows from failure to capture plastics in other traded waste products.^43^^,^^84^ Second, illegal plastic exports are significant, with estimates suggesting that 15%–30% of EU waste trade involves illegal shipment.^85^ Third, plastic waste exported for recycling is frequently mismanaged and may end up in the environment.^86^

Without reliable data on the quantity, composition, and destination of traded plastics and plastic-containing products, governments struggle to enforce existing trade regulations, prevent illegal exports, and ensure environmentally sound management of plastic waste. Incomplete information on plastic trade flows also undermines the ability to assess the effectiveness of policy instruments such as export bans and waste shipment regulations. Moreover, the lack of transparency around hidden plastics in traded goods complicates efforts to allocate responsibility for plastic pollution along global value chains, which in turn weakens accountability.

#### KG 4.4 Social cost of plastics

Social costs broadly refer to the total impact of an activity on societal welfare, combining direct expenses, productivity losses, health impacts, environmental degradation, and other external costs shifted onto the public.^87^ Little research quantifies the full social cost of plastics. Plastics play an important role in most sectors and systems of production and consumption, making it highly challenging to calculate aggregated social costs. Some studies calculate the benefits of reduced, or the costs of increased, plastic pollution,^88^^,^^89^ but no standard methodologies exist.^90^ Some estimates quantify the economic consequences of plastic pollution on activities like fishing, tourism, and shipping.^91^ However, these estimates overlook values of people and places burdened by pollution (e.g., human life, human health, aesthetic values, cultural or religious significance, and recreational value), including variation across geographies and population groups.^80^^,^^91^^,^^92^ On top of these social costs, should be added the costs of current fossil fuel subsidies to plastic production. While estimates demonstrate that subsidies are substantial,^93^ it is highly complicated to assess the overall level of subsidies for fossil-based plastics and consolidated data are unavailable.^91^

Without robust and comprehensive estimates of the social costs of plastic pollution, governments lack the evidence base needed to justify and design effective policy instruments and regulatory interventions. The absence of standardized methodologies also limits the comparability of assessments across countries and over time, potentially reducing the political feasibility of ambitious preventive measures. As a result, the true societal benefits of immediate action may be underestimated, leading to delayed interventions and potentially higher long-term social costs.

## Discussion

To support more effective plastic governance, we identify four strategic action areas that respond to the identified KGs, these include (1) knowledge generation, (2) methodological development, (3) transparency and disclosure, and (4) tracking and traceability. These were derived through an iterative clustering of KGs based on their shared implications for governance and the types of interventions required to address them (see STAR Methods). As such, they represent categories of intervention required to address how KGs constrain effective plastic governance in the EU.

Table 1 outlines each strategic action area, provides a brief description, and indicates the primary KGs targeted. Some KGs are present across multiple strategic action areas. This overlap reflects the cross-cutting nature of plastic governance, where individual KGs can require multiple types of action. For example, the KG related to chemical recycling of plastics cannot be addressed through knowledge generation alone but also hinges on greater transparency and disclosure to make existing knowledge accessible for decision makers. The strategic action areas discussed here are not intended as a comprehensive policy blueprint. Rather, they highlight enabling conditions that repeatedly emerged as necessary for addressing the identified KGs and enhancing plastic governance interventions in the EU.

**Table 1:** Strategic action areas to address identified KGs and enhance plastic governance

Strategic action area	Description	Primary KGs addressed
Knowledge generation	generate new empirical evidence to fill critical gaps and determine thresholds	KG1.1 sustainable levels of production KG3.3 chemical recycling KG3.4 remediation of plastic pollution KG4.1 micro(nano)plastics KG4.2 hazard and exposure KG4.4 social cost of plastics
Methodological development	develop standardized, replicable, and actionable methodologies	KG1.2 material substitution and problem shifting KG2.1 plastic waste prevention efforts KG2.2 essential use KG3.1 plastic waste volumes KG4.1 micro(nano)plastics KG4.2 hazard and exposure
Transparency and disclosure	implement mandatory reporting systems and expand disclosure requirements	KG1.3 chemical composition KG3.2 recycling outcomes KG3.3 chemical recycling KG4.3 plastic trade flows KG4.4 social cost of plastics
Tracking and traceability	develop systems to monitor material flows and establish product tracking mechanisms	KG1.3 chemical composition KG2.1 plastic waste prevention efforts KG3.2 recycling outcomes KG4.3 plastic trade flows

A key insight from this analysis is that while generating new evidence through research is essential in several areas, many of the identified gaps persist due to limitations in data accessibility, lack of standardized methodologies, insufficient transparency, and weak systems for tracking material flows. The strategic action areas therefore reflect the need for a broader set of governance and infrastructure interventions beyond further research, which could all merit attention in the upcoming EU Circular Economy Act, which is currently under preparation.^94^

### Knowledge generation

Fundamental uncertainties exist regarding the effects and costs associated with the ubiquity of plastics in contemporary society. These are evident in the KGs related to chemical and particle hazards (hazard and exposure), social costs (social cost of plastics), and the sources, behavior, and impacts of micro(nano)plastics (micro(nano)plastics), all reflecting limitations in current understanding of harms posed by the plastic system. In parallel, there is uncertainty regarding sustainable levels of plastic production (sustainable levels of production), reflecting limited knowledge about the thresholds we should strive for. Meanwhile, technologies, such as chemical recycling and pollution remediation (remediation of plastic pollution), are developing and being supported despite limited understanding of their real-world performance, environmental trade-offs, or long-term consequences. All these topics points toward possible future research priorities to be emphasized in the upcoming revision of the European Framework Programme (FP10).^95^

A common denominator across these KGs is the limited availability of empirical evidence, which constrains the ability to assess harms, thresholds, and trade-offs. Addressing this limitation is closely tied to the involvement of a broad range of actors, including research institutions and research funders such as public agencies, foundations and the private sector. Universities and research institutions are likely to play a critical role, particularly in advancing fundamental understandings of plastics’ impacts and sustainable alternatives.

When it comes to the assessment of chemical hazards, the challenge is to undertake systematic, large-scale, and sustained research efforts. This calls for dedicated investment to enable hazard assessments of all chemicals used in plastic production, work often outside the scope of traditional academic funding geared toward novelty, but which, nonetheless, is essential for more informed governance.^96^ Given the scale and complexity of this task, strategic prioritization of research is warranted. In particular, focusing on hazard identification rather than full exposure characterization is appropriate in contexts where full risk-based assessments are unlikely to be feasible.^97^ At the same time, the scale and complexity of such assessments should be reduced through “chemical simplification”, both by reducing the number of chemicals used and molecular complexity and by applying grouping approaches that help avoid regrettable substitutions.^98^ While the importance of this was explicitly acknowledged in the EU Chemicals Strategy for Sustainability,^39^ and the European Chemicals Agency has moved to adopt grouping approaches to assessment of chemicals,^99^ the recent decision to abandon a revision of the Registration, Evaluation, Authorization, and Restriction of Chemicals (REACH) regulation raises significant questions regarding future opportunities to not just assess but also regulate chemicals by groups, for example, regarding bisphenols.^100^

Similar challenges arise in the context of recycled plastics, where heterogeneous and insufficiently characterized material streams make comprehensive hazard assessments particularly difficult. Here, governance efforts may be better directed toward stricter material control, such as closed-loop systems or targeted use restrictions.^101^^,^^102^

While these gaps highlight the need for continued investment in research, scientific uncertainty should not be used as a justification for delaying policy action. A substantial body of research shows that uncertainty has historically been mobilized as a deliberate delay tactic by industry actors, whereby scientific doubt is amplified to postpone regulation despite accumulating evidence of harm.^103^^,^^104^ Similar strategies have been documented across environmental domains, including plastic pollution. In such cases, calls for additional research are used to defer decisions and weaken regulatory ambition.^105^^,^^106^ The framing and mobilization of KGs are, thus, shaped by the diverse objectives of stakeholders, some of which may conflict with climate, environmental, and public health goals.^107^^,^^108^ This underscores the importance of strategies to identify and manage conflicts of interest. Recent analyses of the global plastic treaty negotiations underline the urgency of this concern. These studies argue that entrenched axes of power exist, whereby petrostates and certain industry interests act to retain or expand economic and political power and to sustain profits across petrochemical plastic supply chains. These interests are, for instance, advanced through the strong presence of industry representatives at the negotiations and, in some cases, within state delegations, pushing for weakened treaty ambition.^105^^,^^109^^,^^110^ The dynamics provide critical context for interpreting the KGs identified through the expert workshop, highlighting that their resolution depends not only on research capacity, but also on the institutional and regulatory conditions that shape how knowledge is generated, standardized, disclosed, and used in decision-making. An issue was taken up in the following action areas on methodological development, transparency and disclosure, and tracking and traceability.

### Methodological development

Several identified KGs highlight the importance of developing standardized methodologies capable of generating consistent, comparable data to support evidence-based decision-making. Developing robust approaches to assess the essentiality of plastics in different contexts (Essential plastic use) is critical. While the concept of essential use was introduced in the EU Chemicals Strategy for Sustainability^39^ and further elaborated by the Commission,^40^ its continued implementation (including potential expansion from chemicals to plastic products) is uncertain following the abandoned REACH revision. Equally important is advancing techniques to detect and quantify nanoplastics (micro(nano)plastics) and their toxicity (hazard and exposure), without which, it is not possible to accurately assess associated hazards. Similarly, evaluating whether material substitution leads to genuine environmental advantages (material substitution and problem shifting) would benefit from standardized methods. In the context of EU policymaking, the recent decision to cancel negotiations regarding the Green Claims Directive,^111^ arguably represents a step backwards in terms of establishing such standardized methods for evaluating environmental benefits.

Improved methodologies for monitoring progress toward political targets are essential for translating political ambitions into action. Without such tools, it is difficult to credibly inform and support policy target setting or expect meaningful alignment from industry and other stakeholders. For example, when the political ambition in the EU is to increase plastic circularity,^112^ existing methods for quantifying plastic waste must be expanded to account for plastics present in non-packaging and residual waste streams (KG 3.1). Without better waste accounting, both the full scale of the problem and potential progress toward greater circularity remain obscured, as it is not possible to accurately diagnose waste generation or verify whether interventions are delivering real improvements. Similarly, if the political ambition is to prioritize higher value strategies within the waste hierarchy, such as reuse, which extends products’ lifespans, we need to develop a standardized methodology for reporting on reuse so that efforts can be incentivized, recognized, and rewarded (plastic waste prevention efforts). For packaging, a similar ambition is articulated in the EU Packaging and Packaging Waste Regulation,^113^ but the actual development of standardized methodologies is still to be finalized and is expected in future implementing acts. Across the EU, there are examples of member states, regions, and cities experimenting with reuse or preparing for reuse targets, but the lack of a standardized methodology hampers data comparability, limiting the ability to benchmark performance and identify best practice.^114^

### Transparency and disclosure

Lack of transparency underlies several KGs. Notably, there is insufficient disclosure of the chemical composition of plastics (chemical composition), which hampers hazard assessments across the value chain. The systemic opacity of plastic trade (plastic trade flows) raises multiple questions, such as whether current recycling practices support intended climate and environmental goals (recycling outcomes). Similarly, there is limited public access to industry claims in the chemical recycling sector (chemical recycling) Although the recently approved EU-implementing act on mass-balance accounting seeks to strengthen auditability,^115^ questions remain regarding public transparency and access to underlying data, and the legislation has been met with sharply divergent reactions. In addition, public subsidies to plastic production remain largely opaque, despite being one of the more readily quantifiable components of the social cost of plastics (social cost of plastics). These KGs are fundamentally about making existing and new information publicly accessible to enable informed decision-making, consistent with the objectives of the Aarhus Convention, to which all EU member states are parties.^116^

On the topic of trade, regulations under the Basel Convention, including its 2021 Plastic Waste Amendments, formally establish transparency and control requirements for plastic waste exports.^117^ However, inconsistent implementation and regulatory loopholes, such as legacy classifications that omit “hidden” plastics in textiles or synthetic rubbers, often undermine these measures, preventing comprehensive traceability and public access to reliable data on the movement and management of exported plastic waste.^43^^,^^117^^,^^118^ Addressing these shortcomings requires stronger regulation, for instance, an expansion of the basel prior informed consent procedure to include all plastic waste, also the hidden, and currently unregulated, categories. Moreover, improving the harmonized commodity description and coding system (HS) that classifies trade flows according to the function of goods rather than the goods’ materials could improve our understanding of the movement of plastics across the globe.^82^ This could result in more effective efforts aimed at governing flows of plastics, for example, more effective regulation avoiding increased exports of plastics to countries with less well-functioning waste handling systems. Beyond regulatory disclosure, tackling KGs related to illegal trade of plastic waste requires targeted law enforcement and improved international coordination. While commercial interests and administrative burdens should be considered, stronger regulatory intervention is necessary to address persistent enforcement failures.^118^^,^^119^ A recent revision to the EU Regulation on Waste Shipments^120^ seeks to address this challenge through a ban on exporting non-hazardous plastic waste to countries that are not members of the Organisation for Economic Co-operation and Development (OECD) and the establishment of an enforcement group targeting illegal waste shipments by the European Anti-Fraud Office.^121^

In the case of chemical recycling, a complementary strategy is to make public access to verifiable evidence on environmental and climate impacts, as well as technical and economic feasibility, a guiding criterion for future public investment. Transparency could be treated as a precondition for public funding.

### Tracking and traceability

Several of the KGs highlight the importance of strengthening and expanding tracking and traceability across the plastic system. We lack insight into how plastics move through the economy (plastic trade flows); it is often impossible to determine what plastics are made of or contain (chemical composition); we have limited knowledge of where plastics have been used and for how long (plastic waste prevention efforts); and we do not know what happens to a lot of plastic waste once sent for treatment (recycling outcomes).

Faced with these challenges, there is a growing need to develop traceability tools that better support plastic supply-chain governance by making information accessible about products’ origin, material composition, and end-of-life handling.^122^^,^^123^ Different types of traceability innovations are emerging, drawing on both digital and physical technologies,^124^ yet their future development and implementation must overcome significant practical, technical, and political barriers. This creates scope for policy support, including targeted funding and the development of shared data standards.^122^ In the EU context, digital product passports (DPPs) are being introduced for selected priority product groups under the Ecodesign for Sustainable Products Regulation,^125^ including information on products with substances of concern with a negative influence on reuse or recycling. A DPP is a digital record that compiles key information about a product within a defined scope, governed by agreed access and data-management rules and accessed electronically via a unique identifier.^126^ While DPPs are currently being developed for products such as textiles, furniture, tyres, mattresses,^127^ and other plastic products, such as packaging where volumes are high, product lifetimes short, and contributions to plastic pollution are substantial, other approaches than DPP may be more practical or effective, reinforcing this as a strategic action area requiring further attention.

While traceability tools such as DPP primarily focus on individual products and supply chains, more effective governance of plastic will also require system-level tracking. It is necessary to enhance and harmonize statistical reporting on plastics, including data on production volumes, the quantity and types of plastic-containing products placed on the market, trade flows, and waste management practices. Current work to implement common end-of-waste criteria across the EU countries is one step toward allowing such harmonization.^128^ Improved tracking and disclosure mechanisms could support transparent monitoring and enable better policy design, enforcement, and public accountability throughout the plastics system.^129^

### Next steps for plastic governance

Together, our analysis highlights that plastic pollution is a complex, systemic challenge with profound consequences for human health and the environment. While the ongoing treaty negotiations represent a critical opportunity for global action, progress is restrained by the current political deadlock. However, the ability to effectively govern plastics is also constrained by a range of KGs, creating a scope for action both in preparation for future treaty negotiations as well as outside treaty negotiations.

Focusing on the context of the EU, this paper has analyzed critical KGs that impede effective plastic governance across the full life cycle of plastics. Our analysis shows that plastic governance is constrained not only by missing evidence but by systemic deficiencies in how knowledge is generated, standardized, disclosed, and monitored across the plastics’ life cycle, underscoring that addressing KGs requires governance and infrastructure interventions alongside further research. Based on the analysis, we identify four strategic action areas: (1) knowledge generation, (2) methodological development, (3) transparency and disclosure, and (4) tracking and traceability. Coordinated efforts across these areas will be key to enabling sustainable, systemic solutions to the complex challenges posed by plastic pollution.

The EU has greater scope to act in some areas, such as developing methodologies, generating new knowledge, and implementing traceability systems. Other areas, particularly those that require international coordination, such as changes to the Basel Convention, are more challenging and depend on multilateral engagement and the current geopolitical landscape.

While we recognize the difficulty of achieving an ambitious treaty, our analysis demonstrates that meaningful progress can still be made within the EU. Pursuing these strategic action areas strengthens the legitimacy of the EU’s ambition to achieve an international agreement that addresses the entire plastic life cycle; it supports more effective plastic governance and provides a foundation to inform and advance future international negotiations.

### Limitations of the study

This study has several limitations. First, the identification and prioritization of KGs were informed by a structured expert consultation and subsequent author-led analysis, meaning that the findings inevitably reflect the expertise, perspectives, and institutional backgrounds represented in the workshop and subsequent process. Although contributors came from diverse disciplinary and organizational contexts, other expert communities may have emphasized different KGs or governance priorities. Second, the literature analysis was conducted through an expert-informed and purposive review rather than a formal systematic review, prioritizing breadth, policy relevance, and emerging evidence over full reproducibility. Third, the analysis focuses on the EU as a governance context. While many of the identified KGs are relevant also outside the EU, the salience of specific gaps and the feasibility of proposed action areas may differ across regions with different institutional capacities, regulatory traditions, and socioeconomic conditions.

## Resource availability

### Lead contact

Further information and requests for resources should be directed to and will be fulfilled by the lead contact, Fredric Bauer (fredric.bauer@miljo.lth.se).

### Materials availability

This study did not generate new, unique materials.

### Data and code availability

• This study did not generate new research datasets. The analysis was informed by a structured expert workshop and published literature. Workshop notes served as internal documentation to support the authors’ qualitative synthesis and are not publicly available because they contain confidential discussions and information that could identify participants.
• This paper does not report custom code.
• Additional information about the study methodology is provided in the STAR Methods. Further information is available from the lead contact upon reasonable request.

## Acknowledgments

We thank the experts who contributed scholarly input during the January 2024 workshop on plastic governance but declined to co-author this paper: Andrés del Castillo (Center for International Environmental Law), Bahar Koyuncu (Ellen MacArthur Foundation), Beatriz Vidal Legaz (European Environment Agency), and Mari Erlandsen (European Environment Agency). Five other contributors from the European and national public administrations preferred not to be named due to their professional roles and are, therefore, acknowledged collectively. The views expressed in this article are solely those of the authors and do not necessarily reflect the views of the workshop contributors or their institutions. Moreover, S.H.J.M., F.B., T.H., L.J.N., E.D., and T.O. acknowledge funding from the 10.13039/100007633Swedish Foundation for Strategic Environmental Research (Mistra) through the program STEPS—Sustainable Plastics and Transition Pathways. T.H. and T.O. also acknowledge funding from the 10.13039/501100002808Carlsberg Foundation through GreenTraC—Green Transition Policy Centre. P.V.-G. acknowledges funding from the 10.13039/501100004359Swedish Research Council for Sustainable Development (FORMAS) for the project “The plastics pollution challenge: a global social-ecological perspective” (No. 2020-01295). K.S. acknowledges funding from the 10.13039/100007214Velux Foundation through MarinePlastic II. The views expressed in this article are the sole responsibility of the authors and in no way represent the views of the European Commission and its services.

## Author contributions

S.H.J.M. and F.B. conceptualized the paper and with T.H. led the value chain working groups and drafted the manuscript. All other authors contributed to the analysis within the working groups and participated in multiple rounds of manuscript review.

## Declaration of interests

F.B., T.H., E.D., T.O., and K.S. are non-remunerated member of the Scientists’ Coalition for an Effective Plastics Treaty. B.C.A. and P.V.-G. are non-remunerated steering committee members of the Scientists’ Coalition for an Effective Plastics Treaty.

## STAR★Methods

### Key resources table

**Table:** 

REAGENT or RESOURCE	SOURCE	IDENTIFIER
**Software and algorithms**		

Microsoft Word	Microsoft	https://www.microsoft.com/da-dk/microsoft-365/word?market=dk
EndNote	Clarivate	https://endnote.com/

### Method details

This study employed a three-phase mixed-methods approach combining a structured expert consultation conducted through an in-person workshop, a subsequent literature analysis, and collaborative synthesis to identify and characterise knowledge gaps for plastics governance across the life cycles and value chains of plastics. While expert elicitation is often used to estimate uncertainties for formal modeling^130^ related approaches can also be used to explore qualitative issues for which current evidence is limited.^131^ The paper did not involve empirical research on human subjects or the collection of personal data, rather the workshop functioned as a scholarly expert consultation informing a subsequent author-led literature-based analysis.

In the first phase, we convened a transdisciplinary expert workshop during a full day in January 2024. The workshop brought together 24 experts working in Europe from diverse fields including environmental science and policy, economics, and social sciences, representing academic institutions, public policy, and civil society organisations. Experts were invited based on their published research contributions and practical experience in plastics-related policy and governance aiming to capture subject-matter expertise in key topics connected to plastics (ecotoxicology, environmental health, climate change mitigation, industrial change) as well as experts on environmental governance (public and private governance initiatives, policy implementation and evaluation). All workshop contributors were invited to be part of the follow-up process and contribute to this paper. Fifteen contributors accepted and have co-authored the paper and nine contributors declined the invitation. Their contributions are acknowledged in the acknowledgments section where individuals who consented to being named are listed, while others are referred to in aggregate. To provide additional transparency regarding the composition of the workshop contributors, an overview of institutional affiliations and the geographic scope of represented institutions is presented in aggregated form in the table below.

**Table 2:** Institutional and geographic composition of the expert workshop

Dimension	Category	Number of experts
Institutional affiliation	academic institution	11
	applied research institution	2
	EU institutions and agencies	8
	national public administration	1
	civil society organisation	2
Geographic scope of institutions	EU-based	21
	non-EU Europe	3

Prior to the workshop, the invited experts submitted key publications and documents deemed relevant for understanding the current state of plastic governance and key knowledge gaps for effective policymaking on plastics. Based on these submissions, the workshop utilised a structured format to elicit expert knowledge in further identifying knowledge gaps. The workshop was divided into three sessions focusing on key themes emerging from the pre-workshop process: (i) existing initiatives and instruments across issue domains, (ii) data, monitoring, and reporting systems, and (iii) emerging multi-scalar governance approaches. The experts were divided into thematic groups in three sessions, each group aiming to identify knowledge gaps relating to their core expertise. The division into smaller groups was part of aiming to capture a wide variety of perspectives on the issue as well as to reduce the risk for individual dominance during the workshop. Workshop discussions were documented to capture collectively identified themes and knowledge gaps, which served as inputs to the subsequent literature-based analysis conducted by the author team. At the end of the workshop 21 potential knowledge gaps had been identified, which served as a starting point for the subsequent author-led analysis. The identification and framing of knowledge gaps are inherently shaped by the expertise and perspectives represented in the workshop and subsequent analysis, reflecting the expert-informed nature of the approach.

Following the workshop, an expert-informed literature review was conducted to validate and expand upon the identified knowledge gaps with the best available knowledge. The author group was divided into four working groups, each covering knowledge gaps relating to one part of the life cycle of plastics (production, use, end-of-life) as well as one group focused on cross-cutting issues. Three authors focused on production, four on use (five including one author contributing to two groups), four on end-of-life, and four on cross-cutting issues.

The authors reviewed recent peer-reviewed articles within their domains of expertise as well as relevant gray literature from international, policy-relevant organisations to capture practice-oriented evidence and insights from ongoing policy processes. Papers and reports included in the review were identified using a combination of iterative, purposive searches in scholarly databases and institutional repositories (e.g., UN iLibrary and OECD iLibrary) as well as citation tracking. Search strategies were developed iteratively within each working group and guided by the thematic focus of the identified knowledge gaps. As a result, the search process was not exhaustive or conducted using a formal systematic review protocol with predefined search strings and inclusion criteria that enable full reproducibility in the manner of systematic reviews. Instead, it was guided by thematic relevance and expert judgment, reflecting a deliberate trade-off to capture a broad and evolving evidence base across disciplines and source types, including materials not systematically indexed in scholarly databases. The working groups conducted detailed analysis of the literature within their respective themes to characterise knowledge gaps according to their nature, implications for governance, and possible policy responses. This framework enabled us to identify cross-cutting issues and interconnections between different life cycle stages.

The third phase consisted of collaborative synthesis and validation. Working group leaders jointly reviewed the contributions of the working groups to ensure consistency and coherence, leading to the merging of similar knowledge gaps across some life cycle stages and resulting in the list of 13 knowledge gaps presented in the paper. The set of identified knowledge gaps was clustered into four strategic action areas to support synthesis and policy relevance. This clustering was first done by the three working group leaders. The clustering followed an iterative and interpretive approach, in which knowledge gaps were grouped based on their shared implications for governance and the types of interventions required to address them, rather than strictly by life cycle stage. The draft manuscript, including the final list of knowledge gaps and the identified action areas, was circulated twice among all authors for review and refinement. This approach allowed us to comprehensively map the landscape of knowledge gaps and identify strategic action areas while maintaining scientific rigor through multiple validation steps.

References were managed using EndNote (Clarivate), and the manuscript was prepared using Microsoft Word (Microsoft).

### Quantification and statistical analysis

This study did not involve statistical hypothesis testing or quantitative data analysis. The study employed a qualitative, expert-informed research design combining a structured expert workshop, literature analysis, and collaborative synthesis to identify and characterize knowledge gaps for plastics governance. No statistical software was used.
